# Recommendations for the diagnosis and treatment of spontaneous intracranial hypotension

**DOI:** 10.1007/s11547-025-02116-6

**Published:** 2025-10-30

**Authors:** Elisa Francesca Maria Ciceri, Luigi Cirillo, Francesco Causin, Ferdinando Caranci, Alessandra Splendiani, Mario Muto, Mauro Bergui

**Affiliations:** 1 UOC Radiologia Diagnostica E Neuroradiologia Interventistica, Istituto Neurologico C. Besta, Milan, Italy; 2 AOPD UOC Neuroradiologia, Azienda Ospedale Università Di Padova, Padua, Italy; 3 Dipartimento Di Medicina Di Precisione, Università Degli Studi Della Campania “Luigi Vanvitelli”, Naples, Italy; 4 Dipartimento Di Scienze Cliniche Applicate E Biotecnologiche, Università Degli Studi Dell’Aquila, L’Aquila, Italy; 5 Diagnostic and Interventional Neuroradiology Unit, Cardarelli Hospital, Naples, Italy; 6 Department of Neuroscience, Neuroradiological Unit, University of Turin; Azienda Ospedaliera Città Della Salute E Della Scienza Hospital, Turin, Italy; 7 UO Neuroradiologia, IRCCS Istituto Delle Scienze Neurologiche Di Bologna, Bologna, Italy; 8 Alma Mater Studiorum, University of Bologna, Bologna, Italy

**Keywords:** Spontaneous intracranial hypotension, CSF leak, CSF-venous fistula, Imaging diagnosis

## Abstract

Spontaneous intracranial hypotension (SIH) is a syndrome characterized by disabling orthostatic headache, resulting from a reduction in volume of cerebrospinal fluid (CSF) likely caused by a CSF leak. It mainly affects women at working age and is probably underdiagnosed. This protocol aims to present a proposal for a practical approach to the diagnosis and treatment of SIH. After a descriptive section of the clinical manifestations of SIH, we present a step-by-step model of action to confirm its diagnosis and treatment, considering different clinical scenarios. The aim is, therefore, to facilitate clinical decision-making through a systematized and individualized approach, aiming to best interest of the patient.

## Introduction

Spontaneous intracranial hypotension (SIH) is characterized by a decreased cerebrospinal fluid (CSF) pressure, presumably due to CSF leak through a weak point in the dura mater. Limited epidemiological data are available. SIH can involve patients of any age, including children and adolescents, but most frequently affects women older than 35 years. According to Shievink et al., the incidence rate is around 3.7–5 patients per 100,000 person/year [1, 2]. Predisposing factors to SIH development include meningeal diverticula, connective tissue diseases (Marfan syndrome, Ehlers-Danlos syndrome, joint hypermobility, neurofibromatosis, Lehman syndrome, and polycystic kidney disease), spondyloticdural tear, and trivial trauma. Genetic studies screening for associated mutations have returned negative results [3]. SIH should be differentiated from other conditions where the cause of hypotension is known such as CSF shunt, traumatic or post-surgical CSF leaks, and lumbar puncture).

Since the opening pressure of the cerebrospinal fluid can be normal in approximately 30%–60% of SIHs [1, 3], the term "cerebrospinal fluid hypovolemia" has been proposed [4, 5] and cerebrospinal fluid manometry is no longer recommended.

Most cases of SIH are related to a spinal CSF leak. Although the debate is still ongoing, there are three hypotheses to explain the loss of CSF: dural laceration (type 1), meningeal diverticulum (type 2), or venous CSF fistula (type 3). Dural laceration occurs when calcified disk fragment or bone spur tears the dura mater longitudinally, causing a loss of cerebrospinal fluid which collects in the epidural space. Leptomeningeal diverticula can form through the dura at the nerve root or at a junction point with the dural sac and are prone to rupture. When large, they can cause rapid CSF outflow, while in other cases, the leak is slower and increased by Valsalva maneuvers [6]. Perineural diverticula are associated in approximately 80% of cases. Venous CSF fistula occurs when the spinal subarachnoid space is directly drained into a paraspinal vein, bypassing the arachnoid granulations, and resulting in a rapid decrease in cerebrospinal fluid volume.

The cause of SIH remains undetected in up to 28% [7] and, even when imaging demonstrates the leakage, it may not be possible to precisely identify the exact location of the fistula point.

The cerebral caudal displacement with traction/distortion of pain-sensitive nerve endings in the dura mater and its blood vessels is considered the causal mechanism of headache and neurological manifestations of the disease. Brainstem downward displacement may be responsible for tinnitus, muffled hearing, nausea, vomiting, dizziness, cranial nerve palsies, neurocognitive changes, and coma. Venous sinus thrombosis and less likely ischemic stroke can also result from SIH. Microhemorrhages from dural defects may lead to superficial siderosis. Subdural hemorrhages may become symptomatic “per se”. [3, 7–11].

**Fig. 1:**
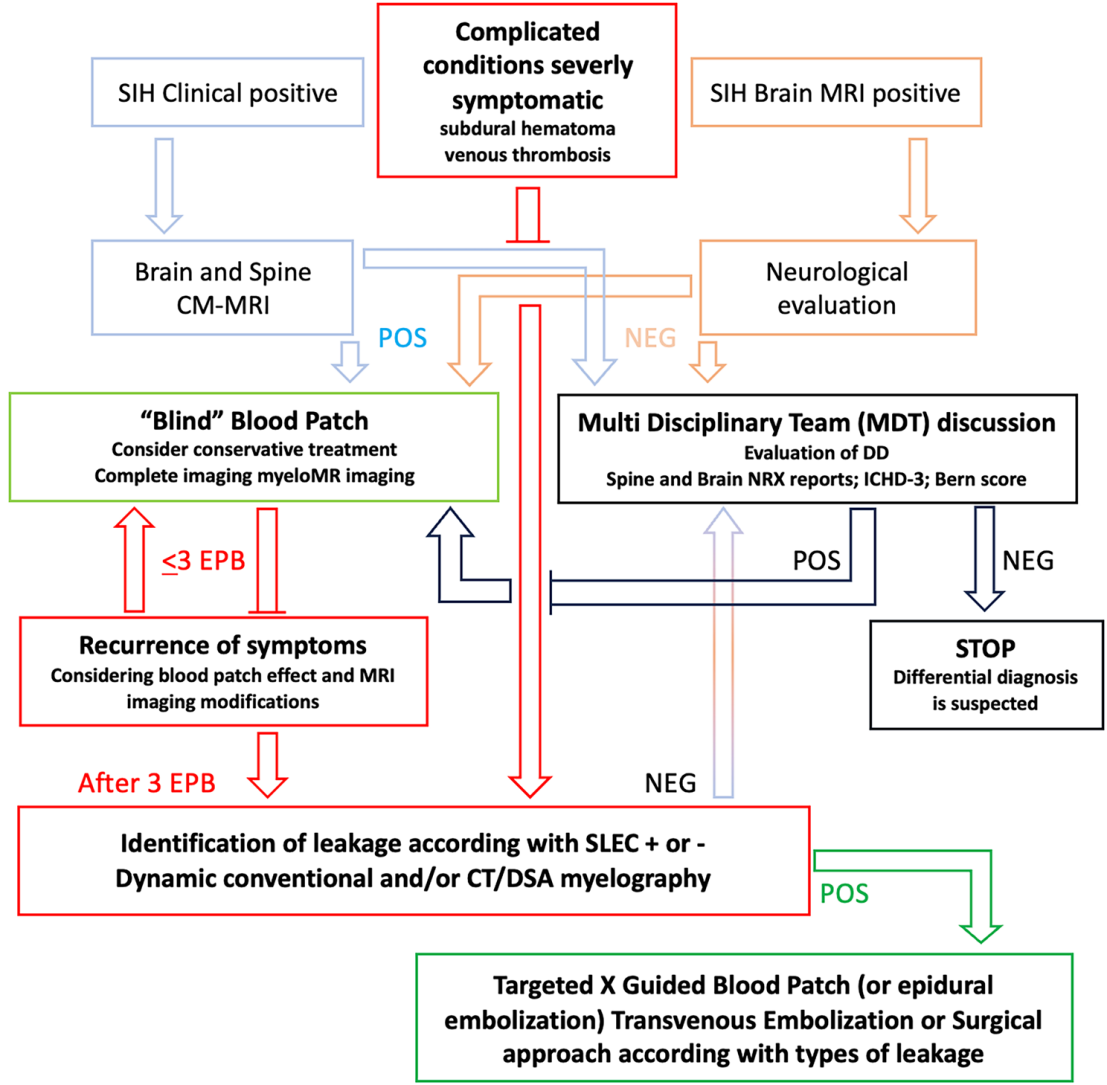
Based on the clinical condition and neuroimaging findings, the proposed flow-chart for management of SIH divides different levels of diagnostic and therapeutic invasiveness. The conditions in the red boxes require greater attention in terms of rapid diagnosis and treatment and the need for an ultra-specialist diagnostic pathway. In green boxes we put the situations with strongest evidence for the recommendation. Finally in black boxes, our suggestions are based on the need to define and train a dedicated multidisciplinary team. Abbreviation: POS positive; NEG negative; EPB Epidural Blood Patch; CM-MRI Myelography-Magnetic Resonance Imaging; SLEC spinal longitudinal epidural collection

Early recognition of SIH is crucial for timely treatment and the prevention of complications. Due to the challenges in diagnosing SIH and understanding its underlying causes, the protocol we propose aims to take a systematic and practical approach to diagnosing SIH (Fig. 1). This will facilitate clinical decision-making and optimize therapeutic outcomes for SIH patients.

## Material and methods

This paper is the result of a joint initiative promoted by a specific group of expert Neuroradiologists under the umbrella of AINR (Italian Association of NeuroRadiology) and Neuroradiology study section of SIRM (Italian Society of Medical and Interventional Radiology).

To organize a practical diagnostic/therapeutic process, two electronic databases (PubMed, and Google Scholar) were searched for SIH from 2000 to June 2024. Keywords like “intracranial hypotension”, “SIH” and “CSF leak” were used. The preferred citations were analysed, and systematic reviews on the SIH were transferred to Mendeley Desktop app version 1.19.3 © 2008–2018. Citation tracking was completed for all the noted articles focusing on the diagnosis and treatment of SIH.

Articles with less than one citation were excluded; therefore, 35 articles were analyzed.

Due to the lack of randomized trials studies, current recommendations are based on observational studies and expert opinion.

We focus on instrumental diagnostic examinations which are essential to confirm diagnosis and to guide treatment. The various treatments proposed were also evaluated in a step-by-step approach, tailored in the face of the comorbidities and the type of patient response over time.

The main findings were summarized in a flow-chart diagram including both diagnosis and treatment.

### SIH Clinical manifestations and differential diagnosis

The main symptom of SIH is orthostatic headache which worsens while standing, immediately, after seconds/minutes, or hours (‘second half of the day headache’) and tends to markedly improve with bedrest [11]. It is generally holocranial or bi-suboccipital but can also be unilateral or pulsatile, mimicking migraine headache. It may worsen with the Valsalva maneuver and tends to resolve after CSF pressure normalization or CSF fistula occlusion. It should be noted that the orthostatic component of the headache tends to attenuate over time, which is why the anamnesis should focus on its initial characteristics. Rarely, the patient may experience a non-orthostatic headache, non-specific, thunderclap (15%), triggered by effort or even with a reverse posture (i.e., worsens with bed rest improving with orthostatism) [12, 13]. In 3% of cases, SIH can occur without any headache [14, 15]. All the above-mentioned factors may contribute to diagnostic challenges with others more common causes of headaches such as primary and secondary headaches. According to the third edition of the International Classification of Headaches [7, 8], the diagnosis of ‘SIH headache’ is "possible" when all the following criteria are present:

1. Any headache associated with CSF hypotension (< 6 cm H20) and/or imaging evidence of CSF fistula;
2. Absence of trauma or procedure that could have caused a CSF leak;
3. Headache that occurs in close temporal relation to CSF hypotension or CSF fluid leak or has led to its discovery;
4. No other diagnosis to explain the headache.

As previously mentioned, lumbar puncture (LP) and CSF manometry are usually not necessary, since CSF opening pressure may be normal [1, 3, 5, 8], suggesting that volume, and not necessarily pressure, maybe a major determinant for the disease [1, 9].

SIH headache is often associated with neck pain/neck stiffness, tinnitus, hearing disorder, photophobia and/or phonophobia, and nausea/vomiting. Less commonly, the patient may present with diplopia (most often due to paresis of the sixth cranial nerve), tremor/parkinsonism, or non-specific complaints. Fatigue and difficulties in concentration (“brain fog”) are extremely common, especially in chronic patients. [15].

Patients may present signs or symptoms related to traction of any cranial nerve, including facial palsy, dysgeusia, or hiccupping [12], cochleo-vestibular manifestations (e.g., unilateral hypoacusia, dizziness, tinnitus, or vertigo), may be due to traction of the eighth cranial nerve. However, these symptoms may also be attributed to alterations of the inner ear perilymph/endolymph pressure. These symptoms are more common among patients older than 45 years [4].

Increase in the body temperature can also be seen in SIH, probable due to inadequacy of the diencephalic thermoregulatory system because of the longitudinal brain-stretching, compression by swollen veins, or by direct cytokines activation, as a result of the damaged blood–brain barrier [16]. In patients with elevated temperature, subarachnoid hemorrhage, and meningitis (viral/aseptic) should be ruled out [15].

Moreover, CSF in patients with SIH may show lymphocytic pleocytosis, which may exceed 200 cells/mm3. Elevated protein concentrations and decreased glucose concentrations in the CSF have also been described in patients with SIH [17, 18]. Symptoms of SIH that may direct the diagnosis toward cerebrovascular diseases include dizziness, balance disorders, numbness or paresthesias of the face or limbs, vision, hearing and taste disorders, and abducens nerve palsy. [19, 20].

It is important to remember that cerebrovenous sinus thrombosis (i.e., cortical vein stroke or sinus thrombosis) can occur more often in patients with SIH. Thus, the diagnosis of venous infarction in patients with SIH can be a diagnostic challenge. [21, 22]. Another unusual presentation of SIH is non-convulsive status epilepticus or isolated seizures in the advanced stages of SIH. Therefore, SIH should also be considered in patients with seizures of unclear etiology or patients presenting epileptiform EEG anomalies but lack of response to antiepileptic drugs [3].

Differential considerations for headache in patients with SIH include tension-type headache, migraine, cervical or occipital headache, cervical radiculopathy, headache attributed to Chiari malformation type I, headache attributed to somatisation disorder, and cough headache. Furthermore, suspicion of SIH requires differentiation from postural orthostatic tachycardia syndrome (POTS) and orthostatic hypotension [16]. Accompanying symptoms may include dizziness, balance, vision, and consciousness disorders. Early initial verification of the diagnosis of both conditions is recommended. [23].

Patients with SIH-related headaches often experience reduced activity during the day because the symptoms tend to worsen in the second half of the day. This can be misinterpreted as typical migraine headaches that worsen with physical exertion [24]. Headaches attributed to SIH may also be misdiagnosed as vestibular migraine because of the common co-occurrence of balance disorders or dizziness that affects about half of the SIH patients. The differentiating feature from headache due to Chiari type I malformation is the duration of the pain. The headache in Chiari type I malformation is brief and typically lasts up to 5 min, as emphasized in the diagnostic criteria, whereas headache from SIH is usually chronic and lasts for hours. In both cases, the headache typically localizes in the occipital location and can be aggravated by coughing, Valsalva maneuver, or anything that increases the intra-abdominal pressure [16]. Once again, it is neuroimaging that helps avoid misdiagnosis by highlighting the features of SIH or Chiari type I.[25].

Finally, less frequent clinical manifestations of SIH can include galactorrhoea, diabetes insipidus, superficial siderosis, or movement disorders such as parkinsonism, ataxia, postural tremor, and chorea, brachial amyotrophy and/or behavior/personality changes [26–29].

As already mentioned, SIH can have several complications, some of which are potentially life-threatening, including subdural hematoma (the most common, in 20% to 25% of cases), cerebral venous thrombosis, uncal herniation, and in the most severe cases brainstem ischemia and coma.

In conclusion, a detailed clinical history—especially the presence of a characteristic headache at disease onset—combined with a thorough understanding of the associated symptoms, forms the cornerstone of clinical diagnosis.

### Diagnostic approach to SIH

Gadolinium-enhanced brain and spine allow diagnosis in 80% of the cases [4].

Findings suggestive of SIH include: diffuse pachymeningeal enhancement (73–80%), subdural collections along the convexities (60% hygroma, 40% hematoma), venous engorgement (57%), enlarged pituitary gland (38%), venous dilatation, and brain sagging (43%) [4, 24, 27, 30] decreased optic nerve diameter and thickness on coronal T2-weighted sequences (> 5% decrease while standing at Transorbital US) [31].

**Fig. 2:**
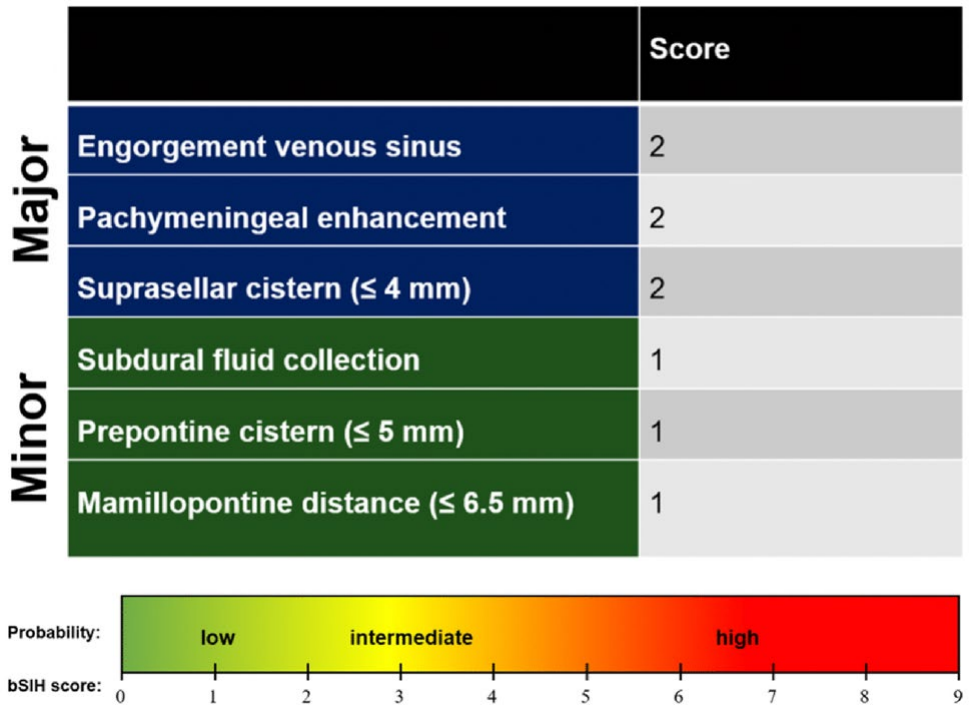
The BERN score is calculated by summation of three major criteria (2 points each) and three minor criteria (1 point each). Patients with a score of ≤ 2, 3–4, or ≥ 5 have a low, intermediate, or high probability of spinal CSF loss, respectively [33]

The Bern score (Fig. 2) is a predictive score based on brain MRI findings in individuals suspected of having spontaneous intracranial hypotension. It categorizes patients into high, intermediate, or low likelihood of detecting a spinal CSF leak or CSF-venous fistula during myelography [32–34].

MRI findings vary according to disease progression time [31, 35]. However, around 20% of SIH cases present normal brain MRI. If the clinical suspicion of SIH remains high, a full spine MRI should be performed, which allows the identification of indirect signs, such as pachymeningeal enhancement, dilated nerve root sheaths, engorged epidural venous sinuses, or meningeal diverticula [2–9, 13–17, 30–37]. At the same time, direct signs of spinal extradural CSF collections (SLEC) (> 50%) should be investigated. Consequently, when SIH is considered, spinal MRI should always be performed simultaneously with the brain MRI [32]. Currently, there is no universally shared spinal MRI protocol for the diagnosis of spontaneous intracranial hypotension (SIH). To address this variability, we propose a standardized spinal MRI protocol developed in collaboration with the Italian Society of Neuroradiology (AINR) emphasizing 3D heavily T2-weighted myelographic sequences like a guide to myelography for detecting and localizing leaks. The proposed protocol is made available on the official AINR website to promote its dissemination and adoption in clinical practice.

(https://ainr.it/ipotensione-liquorale-ipertensione-endocranica-proposta-di-standard-minimi-per-esami-neuroradiologici/).

**Fig. 3:**
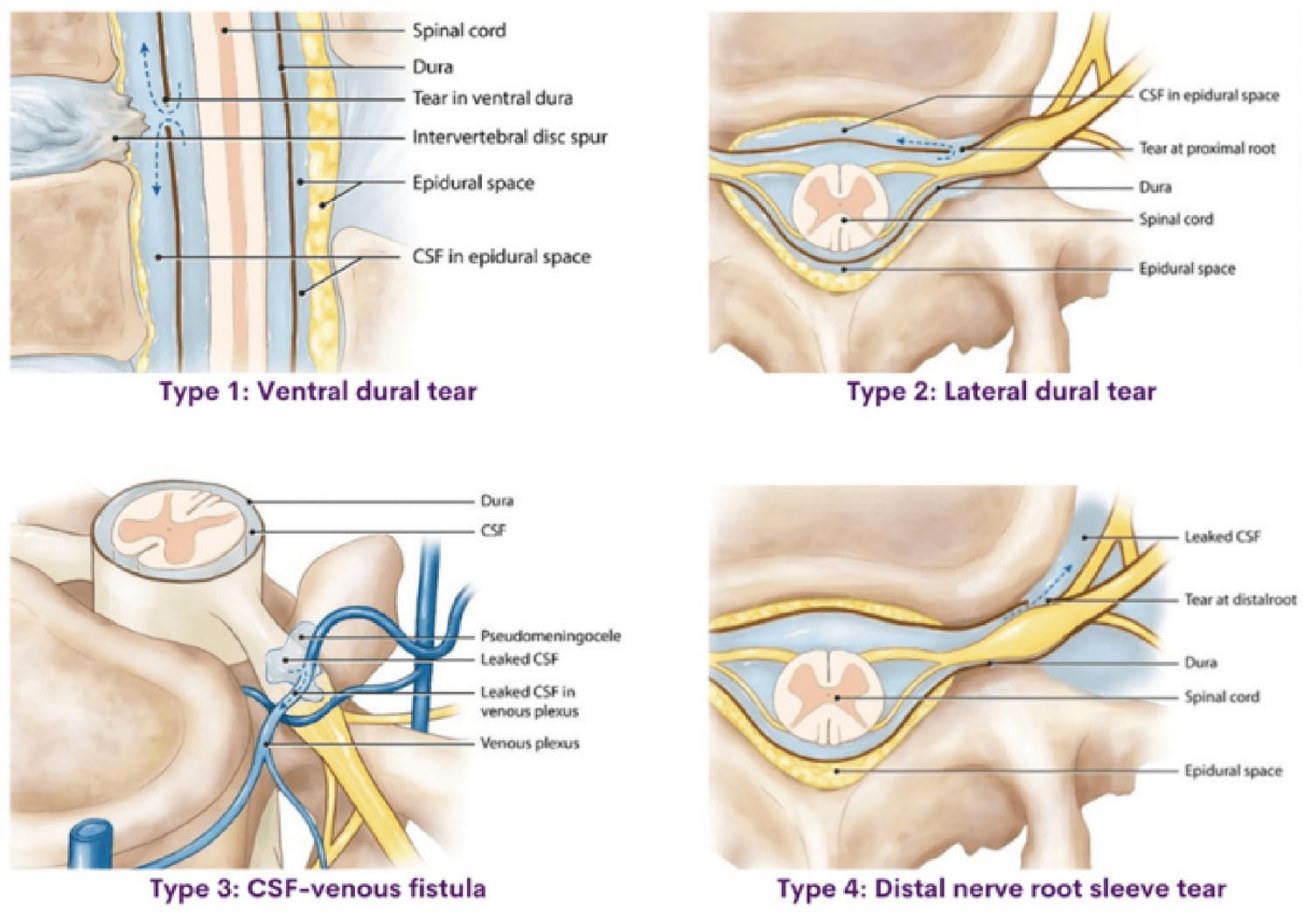
Spinal MR imaging could dichotomize patients with SIH into SLEC-P and SLEC-N populations and determine the nature of their underlying CSF leak and finally prescribe the positioning (prone, supine, or lateral decubitus) for subsequent dynamic myelogram studies [30]

The exact location and type of the CSF leak, according to Farb classification (Fig. 3) can be assessed by isotopic cisternography, which more recently has been supplanted by myelography techniques, with a better spatial resolution [27, 32, 38]. These neuroimaging techniques present a sensitivity that range from 48 to 76%, in detecting the site of CSF leak [5, 32]. Among them, MRI myelography with gadolinium seems the most sensitive, 75%, but the reported cases in the literature are limited to only 87 patients [4]. Interestingly, a recent meta-analysis [37], including 643 patients, found no differences in diagnostic performance between myelo-MRI with intravenous (86%) or intrathecal administration of gadolinium (86% versus 83%). Conventional digital subtraction myelography better enables the detection of type 2 CSF leaks [4]. Intrathecal contrast-enhanced CT myelography is easier to perform and is now the first choice, particularly if MRI myelography results normal. Dynamic myelography or CT myelography performed with flat panel cone beam-CT, which represents its evolution, seems to be the best option for studying high-flow leaks (type 3), often not detected with other methods[6, 39]-(GIF).

### Treatment

To date, there are no randomized controlled studies that support SIH treatment. Therefore, current recommendations are based on observational studies and expert opinion.

*First option* in case of uncomplicated and mildly symptomatic SIH can be conservative management: bed rest, reinforcement of oral hydration, and simple analgesia (paracetamol, non-steroidal anti-inflammatory drugs, caffeine 200 to 300 mg 2 – 3 id PO). The real benefits and therapeutic effects of this choice are controversial. Furthermore, due to the delay in diagnosis, some patients have already used empirical conservative treatment methods before hospital admission (i.e., bed rest and simple analgesia at home) [1].

*Second option*, if there is no clinical improvement despite conservative strategy, or the patient is severely symptomatic, a non-targeted epidural blood patch (EBP) should be considered. This technique consists of the injection of more than 20 mL of autologous blood (collected by venous puncture under aseptic conditions) in the epidural lumbar space, without the need to identify the location of the CSF leak. Fibrin glue and contrast can be added to better stabilize the result and to safely verify the progression of the patch, if done under fluoroscopy [29]. EBP has a few contraindications, including anticoagulant regimen and infections. Similar to any mini-invasive procedure, it is considered safe, with minor and self-limited adverse effects, such as lumbar or radicular pain, dizziness, paraesthesia and, in rare cases, transient bradycardia. Usually, the patient remains bed-rested for 1–2 h after the procedure. The rapid relief of complaints results from the increased CSF pressure (caused by the epidural iatrogenic ‘hematoma’) and the eventual formation of a fibrin clot at the level of the dural defect, which seal the CSF leak. [1–3, 29, 34]. According to clinical presentation, the procedure should be performed in day-hospital admission, and the patients should be re-evaluated with an early follow-up. If the headache has early resolved or has improved substantially (mild and non-disabling headache), clinical and instrumental monitoring should be maintained for some months. Moderate to intense physical exertion should be avoided during this period.

A first blind EBP is effective in about two-thirds of patients [28, 38]. If there is recrudescence or lack of improvement in the headache, a second EBP should be performed days or weeks later.

Observational studies have demonstrated that the majority of patients achieved symptomatic relief after one or two blood patches, and this effect may be cumulative [38]. Some authors also suggest carrying out of a third undirected lumbar blood patch, if there is partial relief of symptoms with the first two procedures.

Emergency blood patch or epidural or intrathecal saline infusion was demonstrated being effective in patients with acute, severe symptoms [40, 41].

*The third option*, in case of no improvement after two or three "blind" EBPs, is to identify the precise site of the CSF leak with direct spinal imaging. [23, 25–29, 31]. In cases where a myelo-MRI study has not yet been carried out, it should be the initial choice; If a CSF leak point is not identified by myelo-MRI, a dynamic myelo-CT or myelography by digital subtraction (conventional) can be performed. If a CSF leak is found, a targeted epidural blood patch guided by computed tomography-CT/fluoroscopy should be considered. In the absence of a favorable clinical response, a second directed blood patch or a neurosurgical intervention (microsurgical repairof CSF fistula, or clipping/closure of meningeal diverticulum) may be considered [31, 38, 39, 42].

In the case of a CSF-venous fistula, embolization [43, 44] or surgery may occlude the leak.

The importance of first treating the CSF leak should be emphasized before addressing complications, particularly in the case of epiphenomenal cerebral venous thrombosis and subdural hematoma, even at risk of worsening them. The multidisciplinary discussion for an individualized approach assumes particular preponderance in those complicated SIH. [10, 12, 31, 42, 45, 46].

Regardless of the treatment method chosen, rebound intracranial hypertension may occur in up to 27% of cases. This complication of treatments must be suspected if there is a change in the characteristics of SIH headache, becoming worse lying down and improving withstanding. Being generally transient, there may be a need for pharmacological treatment (with acetazolamide, in most cases) [44, 47].

Finally, based on what described and discussed, our proposal for diagnostic action and therapy in the case of SIH is summarized in Fig. 1.

## Conclusion

The diagnosis of SIH is not always straightforward. Given the severity of symptoms and the potential complications, it is crucial to initiate appropriate treatment promptly. This protocol offers a step-by-step approach, guiding clinicians from diagnosis confirmation to symptomatic management and, when necessary, targeted treatment based on the most relevant complementary examinations. Our approach aims to streamline the clinical decision-making process in SIH cases through a systematic and personalized strategy, ensuring that the patient’s best interest is always the priority.
